# Cynical hostility increases whereas sense of coherence decreases the odds for current suicidal thoughts: A cross‐sectional study of the general adult population sample

**DOI:** 10.1002/hsr2.1464

**Published:** 2023-07-28

**Authors:** Raimo Palmu, Timo Partonen

**Affiliations:** ^1^ Department of Psychiatry University of Helsinki and Helsinki University Hospital Helsinki Finland; ^2^ Department of Public Health and Welfare Finnish Institute for Health and Welfare Helsinki Finland

**Keywords:** national, suicidal ideation, suicide attempt, survey

## Abstract

**Background and Aims:**

Earlier, somatic diseases and mental disorders have been associated with cynical hostility as well as sense of coherence, but there is a gap of knowledge, whether they contribute to suicidality at population level.

**Methods:**

A random sample of adults, representative of the general population living in Finland, participated in a nationwide health examination study. For 4387 participants aged 18–97 years, we analyzed, after controlling for confounding factors, whether cynical hostility, as assessed with the 8‐item Cook‐Medley Hostility Scale, or sense of coherence, as assessed with the 13‐item Sense of Coherence Scale, contributed to current suicidal thoughts during the past 7 days as scored on the 25‐item Hopkins Symptom Checklist.

**Results:**

Suicidal thoughts (current thoughts of ending one's life) were associated significantly with cynical hostility (*p* < 0.001) as well as with sense of coherence (*p* < 0.001). Of the specific items of cynical hostility, the item “I am sure that most people do not have problems with lying for their own good” was associated most strongly with current suicidal thoughts (*p* < 0.001).

**Conclusion:**

Cynical hostility predicted current suicidal thoughts in a population‐based sample of adults aged 18–97 years. Sense of coherence protected from current suicidal thoughts.

## 1 BACKGROUND AND AIMS

Cynical hostility is an individual disposition comprising of affective, cognitive, and behavioral components. It links to health‐related problems presenting somatic and psychological symptoms,^1^ cardiovascular risks and mortality,^2^, ^3^, ^4^ as well as risk‐taking behaviors,^5^ among which suicidality, i.e., suicide ideation as well as suicide attempts, is life‐threatening. Suicide ideation is either active or passive. Active suicide ideation includes thoughts about taking action to end one's life, including identifying a method, having a plan, or having intent to act, whereas passive suicide ideation comprises of thoughts about death or wanting to be dead without any plan or intent.^6^ Suicide attempt is a potentially self‐injurious behavior associated with at least some intent to die.^6^ Earlier, no difference in measures of hostility nor in most measures of the severity of depression was found between patients with major depression with or without suicidal ideation, but the suicidal patients did demonstrate greater cynicism.^7^

Sense of coherence, or to which extent the individual perceives the world as comprehensible, manageable, and meaningful, is an individual disposition reflecting the capacity to respond to and resolve challenging or even life‐threatening situations.^8^ It links to the self‐rated status of health and especially that of mental health.^9^ Sense of coherence might counterbalance cynical hostility, and therefore subsequently suicidality as well.

With conducting a systematic literature search, we did not find reports on the relationship of cynical hostility with suicidality. However, in a nationwide health examination survey representative of the general adult population living in Finland, a high‐income Nordic country with approximately 5.55 million inhabitants, cynical hostility as well as sense of coherence were assessed with validates scales. Therefore, the aims of our study were to analyze whether cynical and hostile dispositions were associated with suicidal thoughts and suicidal ideation as well as suicide attempts, and if they were, to assess the magnitude of these associations.

## 2 METHODS

The Health 2011 Survey^10^ was a follow‐up study of the Health 2000 Survey.^11^ The invitation to take part in the Health 2011 Survey was sent to all persons (aged 29 or older in 2011) who had been included in the random sample of the national Health 2000 Survey (*n* = 8177) and were still living in Finland, as well as to a new random sample of persons aged 18–28 years (*n* = 1994). The data were collected in 60 locations nationwide between August 2011 and June 2012. The Health 2011 Survey is considered as a representative of the adult population living in Finland in 2011.

The study was reviewed and approved by the Coordinating Ethics Committee of the Hospital District of Helsinki and Uusimaa (HUS, reference 45/13/03/00/11). The study protocol followed the Declaration of Helsinki and its amendments. After complete description of the study to the subjects, written informed consent was obtained.

### 2.1 Assessment of cynical hostility

Cynical hostility was assessed with the 8‐item scale^12^, ^13^ as modified from the original Cook‐Medley Hostility Scale.^14^ The participants responded on the ordinal scale (1–4) to the following items: (1) I am sure that most people do not have problems with lying for their own good; (2) Most people are good and honest mainly because they are afraid to get caught; (3) Most people are ready to use any means, also dishonest ones, to gain benefits; (4) I often think what could be the real reasons when others do something for my benefit; (5) Nobody cares much about what happens to somebody else; (6) It is better not to trust anyone; (7) Most people make friends because they think friends can be useful for their purposes; and (8) Most people would not want to go through the trouble to help other people. The total score ranged from 8 (high hostility) to 32 (low hostility).

### 2.2 Assessment of sense of coherence

Sense of coherence was assessed with the 13‐item scale (SOC‐13)^15^ as modified from the original 29‐item scale.^8^ The participants responded on the ordinal scale (1–7) to the following items describing meaningfulness, comprehensibility, and manageability: (1) Until now your life has had (“No clear goals or purpose at all” to “Very clear goals and purpose”); (2) Do you have the feeling that you do not really care about what goes on around you? (“Never happened” to “Always happened”); (3) Has it happened in the past that you were surprised by the behavior of people whom you thought you knew well? (“Never happened” to “Always happened”); (4) Has it happened that people whom you counted on disappointed you? (“Never happened” to “Always happened”); (5) Do you have the feeling that you are being treated unfairly? (“Very often” to “Very seldom or never”); (6) Do you have the feeling that you are in an unfamiliar situation and do not know what to do? (“Very often” to “Very seldom or never”); (7) Doing the things you do every day is (“A source of deep pleasure and satisfaction” to “A source of pain satisfaction and boredom”); (8) Do you have very mixed‐up feelings and ideas? (“Very often” to “Very seldom or never”); (9) Many people, even those with a strong character, sometimes feel like sad sacks (losers) in certain situations. How often have you felt this way in the past? (“Never” to “Very often”); (10) When something happened, have you generally found that (“You overestimated or underestimated its importance” to “You saw things in the right proportion”); (11) How often do you have the feeling that there is little meaning in the things you do in your daily life? (“Very often” to “Very seldom or never”); (12) How often do you have feelings that you are not sure you can keep under control? (“Very often” to “Very seldom or never”); and (13) Does is happen that you have feelings inside you would rather not feel? (“Very often” to “Very seldom or never”). Five items were reverse coded (items 2, 3, 4, 7, and 9). A total score ranged from 13 (weak sense of coherence) to 91 (strong sense of coherence).

### 2.3 Assessment of suicidality

The item 23 of the 25‐item Hopkins Symptom Checklist (HSCL‐25),^16^ a screening instrument for symptoms of depression and anxiety, asks “How much have thoughts of ending your life bothered you during the past 7 days?” The response alternatives were “Not at all,” “A little,” “Quite a bit,” or “Extremely.” A participant was defined as non‐suicidal if the answer to this question was “Not at all”, whereas those, who gave any other answer to this question, were defined as suicidal. These data were derived from 4387 participants aged 18–97 years.

In addition, the participants aged 18–28 years were asked two questions on suicidality. First, “Have you ever seriously thought about committing suicide.” The response alternatives were “Never,” “Yes, last time was less than 6 months ago,” “Yes, last time was from 6 to 12 months ago,” or “Yes, last time was more than 12 months ago.” Second, “Have you ever attempted suicide, planned or unplanned.” The response alternatives were “Never,” “Yes, once,” or “Yes, more than once, altogether [the number of] times.” A participant was defined as non‐suicidal if the answer to both questions was “Never,” whereas those, who gave any other answer to either question, were defined as suicidal. These data were derived from 802 participants.

Each health examination was scheduled to take about 4 h, and the health examinations did take from 3.5 to 6 h. Feedback received from the respondents on the data collection was mainly positive, and that it was easy to participate in the health examination. In the beginning of the health examination, the questionnaire in which suicidal ideation and suicide attempts had been asked was checked by the study nurses, and if any alarm of suicide risk emerged, it was acted on, with their study team including a medical doctor, as appropriate. The study nurses conducting the health examinations and interviews could in addition enter any relevant notes and comments in the remark field of the program used for data collection. The questionnaire in which suicidal thoughts were asked as well as a feedback questionnaire on their experiences of the health examination were given to the participants for completion either during the health examination or at home within 2 weeks using an electronic questionnaire on the Health 2011 study website. After reception, the data from the questionnaires were checked by the study personnel and acted on, if indicated, as appropriate.

### 2.4 Statistical analysis

Descriptive data were calculated, tested with the Pearson Chi‐squared test or Fisher's exact test, as appropriate, and reported. Partial correlation analysis, adjusted for age, gender, university hospital catchment area covering the geographical area of the country, and level of education, was calculated to yield the Spearman's rank correlation coefficient of the cynical hostility total score with the SOC‐13 total score. The differences in measures of interest between the two groups (suicidal vs. non‐suicidal) were tested with non‐parametric (Mann–Whitney *U* test) or parametric (the Student *t*‐test) tests, as appropriate based on the normality of data distribution. By using principal component analysis, the 8 items of cynical hostility were calculated together with an indicator of suicidality (suicidal thoughts, suicidal ideation, suicide attempts) for sensitivity analysis, as well as for estimation of the magnitude of the association. The Cronbach's alpha‐values of 0.86–0.74 indicated good reliability for each set of variables in all these three principal component analyses. In the binary logistic regression analysis, the variable of current suicidal thoughts was tested as the dependent variable, and the cynical hostility total score, the items of cynical hostility and the total score for sense of coherence were tested as the explanatory variables one by one. The independent contributions of the eight items of cynical hostility to current suicidal thoughts were tested finally with the forward stepwise multivariate regression analysis. For all these models, age (in years), gender (female or male), university hospital catchment area covering the geographical area of the country (Helsinki, Turku, Tampere Kuopio, or Oulu, which altogether cover the geographical mainland area of the country), and level of education (basic, middle, or high) were controlled, and the independent contributions of each explanatory variable were reported. The level of significance was a priori set at *p* < 0.05, and the calculated tests were two‐sided.

The SURVO MM software (Survo Systems, Helsinki, Finland) was used for the factor analysis, and the IBM SPSS Statistics for Windows, version 29, software (International Business Machines Corporation) for the remaining statistical analyses.

## 3 RESULTS

Descriptive data of the sample by suicidality (HSCL‐25) are presented in Table 1. There was a reverse correlation between the total score of Cook‐Medley Hostility Scale and the SOC‐13 total score (*rho*‐value of +0.472, *p* < 0.001).

**Table 1:** Sample characteristics by current suicidal thoughts (HSCL‐25).

	Suicidality	No suicidality	*p*
Gender, *n* (%)			0.64
men	54 (2.8)	1866 (97.2)	
women	63 (2.6)	2404 (97.4)	
Age, mean (*SD*)	57.4 (17.2)	55.1 (14.7)	0.178
Cynical hostility, total score, mean (*SD*)	18.4 (4.6)	23.7 (4.5)	<0.001
Sense of coherence, total score, mean (*SD*)	55.3 (13.3)	73.3 (10.2)	<0.001
HSCL‐25, total score, mean (*SD*)			
HSCL‐25, anxiety, mean (*SD*)	1.7 (0.5)	1.2 (0.2)	<0.001
HSCL‐25, depression, mean (*SD*)	2.2 (0.6)	1.3 (0.3)	<0.001
HSCL‐25, somatic symptoms, mean (*SD*)	2.0 (0.5)	1.2 (0.3)	<0.001

Abbreviations: HSCL‐25, 25‐item Hopkins Symptom Checklist; *SD*, standard deviation.

All the items of cynical hostility were significantly associated with current suicidal thoughts (Table 2). Further, for the participants aged 18–28 years in specific, all the items of cynical hostility were significantly associated with suicidality based on the two direct questions (suicidal ideation and suicide attempts), except “Most people are good and honest mainly because they are afraid to get caught” (Table S1).

**Table 2:** Cynical hostility (Cook‐Medley) items by current suicidal thoughts (HSCL‐25).

	Suicidality	No suicidality	*p*
Cynical hostility, items	Mean (*SD*)	Mean (*SD*)	
I am sure that most people do not have problems with lying for their own good.	1.94 (0.80)	2.57 (0.76)	<0.001
Most people are good and honest mainly because they are afraid to get caught.	2.43 (0.77)	2.66 (0.86)	0.003
Most people are ready to use any means, also dishonest ones, to gain benefits.	2.15 (0.70)	2.76 (0.77)	<0.001
I often think what could be the real reasons when others do something for my benefit.	2.44 (0.87)	3.23 (0.78)	<0.001
Nobody cares much about what happens to somebody else.	2.94 (0.90)	3.06 (0.74)	<0.001
It is better not to trust anyone.	2.34 (1.01)	3,23 (0.81)	<0.001
Most people make friends because they think friends can be useful for their purposes.	2.41 (0.84)	3.15 (0.77)	<0.001
Most people would not want to go through the trouble to help other people.	2.40 (0.81)	2.97 (0.76)	<0.001

Abbreviations: HSCL‐25, 25‐item Hopkins Symptom Checklist; *SD*, standard deviation.

According to the binary regression models, the HSCL‐25 item 23 (“Current thoughts of ending one's life”) was associated significantly with the cynical hostility total score (*p* < 0.001), with all the items except that of “Most people are good and honest mainly because they are afraid to get caught,” as well as with the SOC‐13 total score (*p* < 0.001; see Table 3). In the forward stepwise multivariate regression analysis, the associations of the cynical hostility items with current suicidal thoughts remained significant for the items of “It is better not to trust anyone,” “I often think what could be the real reasons when others do something for my benefit,” “I am sure that most people do not have problems with lying for their own good,” “Most people are good and honest mainly because they are afraid to get caught,” and “Most people make friends because they think friends can be useful for their purposes” in this rank order.

**Table 3:** Odds ratios of cynical hostility and sense of coherence for current suicidal thoughts (HSCL‐25).

	Suicidality			
	Beta	OR	95% CI	*p*
Cynical hostility, total score	−0.240	0.787	0.751–0.823	<0.001
I am sure that most people do not have problems with lying for their own good.	−1.054	0.348	0.268–0.453	<0.001
Most people are good and honest mainly because they are afraid to get caught.	−0.216	0.806	0.643–1.010	0.061
Most people are ready to use any means, also dishonest ones, in order to gain benefits.	−0.924	0.397	0.309–0.510	<0.001
I often think what could be the real reasons when others do something for my benefit.	−1.009	0.364	0.292–0.455	<0.001
Nobody cares much about what happens to somebody else.	−0.979	0.376	0.294–0.479	<0.001
It is better not to trust anyone.	−1.041	0.353	0.286–0.436	<0.001
Most people make friends because they think friends can be useful for their purposes.	−1.035	0.355	0.281–0.448	<0.001
Most people would not want to go through the trouble to help other people.	−0.869	0.419	0.326–0.539	<0.001
Sense of coherence, total score	−0.123	0.884	0.868–0.900	<0.001

Abbreviations: CI, confidence interval; HSCL‐25, 25‐item Hopkins Symptom Checklist; OR, odds ratio.

In the principal component analysis, the HSCL‐25 item 23 (“Current thoughts of ending one's life”) loaded at its strongest (loading value of −0.176) on the same factor with the cynical hostility item “Most people would not want to go through the trouble to help other people” (loading value of +0.709; see Table S2).

Concerning the participants aged 18–28 years, the direct question on suicidal ideation loaded at its strongest (loading value of −0.564) on the same factor with the cynical hostility item “It is better not to trust anyone” (loading value of +0.647; see Table S3). Suicidality as assessed with the two direct questions (suicidal ideation and suicide attempts) loaded at its strongest (loading value of −0.495) on the same factor with the cynical hostility item “It is better not to trust anyone” (loading value of +0.661; see Table S4).

## 4 DISCUSSION

Our main finding was that current suicidal thoughts were robustly associated not only with cynical hostility, but also with sense of coherence. Further, sense of coherence showed a reverse correlation with current suicidal thoughts as well as with cynical hostility. We did not find any earlier report of associations of suicidality with cynical hostility or sense of coherence from studies being representative of the general population. Instead, sense of coherence in relation to suicidality has been examined earlier in selected sub‐populations such as military conscripts in Norway, Greece, and Finland.^17^, ^18^, ^19^

In earlier reports cynical hostility has been associated with a range of somatic health problems,^1^, ^2^, ^3^, ^4^ and sense of coherence with mental health status as a positive resource.^9^ We corroborated this view with our finding from a nationwide random sample of adults representative of the general population that there was a significant reverse correlation between the total score of cynical hostility and the total score of sense of coherence. Furthermore, the odds ratios of cynical hostility as well as sense of coherence for suicidality were both independently significant but reverse in their direction.

To our knowledge, we were able to deliver for the first time the representative quantitative estimates of the influence of cynical hostility as well as that of sense of coherence on suicidality at the level of general adult population. From the published literature, we retrieved only two studies in which both cynical hostility and sense of coherence were assessed simultaneously. On the one hand, both high levels of cynical hostility and low levels of sense of coherence were associated with poor mental health related quality of life among patients with coronary heart disease.^20^ On the other hand, religious motivation in relation to cardiovascular reactivity among middle‐aged adults was investigated, but the relationship of cynical hostility with sense of coherence was not analyzed.^21^ Neither of these studies focused on suicidality.

Potential implications from our study are for suicide prevention. Any positive downward trend in suicide mortality cannot be expected to continue without specific measures preventing further suicides. Suicide mortality can be expected to gradually increase due to population growth, and if the current suicide prevention measures lose their effectiveness, increases in the number of deaths from suicide might emerge in any population. Therefore, it is essential that novel means for identifying the risk of suicide are being investigated at large, and both risk and protection factors are being estimated. Here, we responded to this challenge and delivered population‐based data on one risk factor and one protection factor that have implications for suicide prevention.

Identification of risk or protection factors of suicide in primary care as well as in specialized somatic care would benefit from, e.g., the assessment of cynical hostility together with that of sense of coherence with the routine usage of these structured and short self‐report forms. Interpretation of the scores on them could thereafter help start conversations with patients, lead to clinical attention and improved care, and subsequently reduce suicidal thoughts.

However, our study has limitations. It was partly based on self‐reported data which were collected with a household interview and a set of questionnaires. In addition, our study design was a cross‐sectional one, so we were not able to analyze the causal relationships. To balance, there are strengths of our study as well. It was based on a population‐based random sample of adults, and the sampling strategy produced a representative sample of the general population living in Finland.

## 5 CONCLUSION

We demonstrated that personal dimensions in form of cynical hostility and sense of coherence had robust associations with current suicidal thoughts, the former one being a risk factor and the latter one being a protection factor.

## AUTHOR CONTRIBUTIONS

**Raimo Palmu**: Conceptualization; Data curation; Formal analysis; Writing—original draft; Writing—review & editing. **Timo Partonen**: Conceptualization; Data curation; Formal analysis; Writing—original draft; Writing—review & editing.

## CONFLICT OF INTEREST STATEMENT

The authors declare no conflicts of interest.

## TRANSPARENCY STATEMENT

The lead author Timo Partonen affirms that this manuscript is an honest, accurate, and transparent account of the study being reported; that no important aspects of the study have been omitted; and that any discrepancies from the study as planned (and, if relevant, registered) have been explained.

## Supporting information

Supporting information.

## Data Availability

The data that support the findings of this study are not publicly available due to ethical, privacy and legal restrictions. If you need further information about the data and its utilization, please contact: terveys‐2000‐2011 (at) thl.fi.
