# Supplementary material for: Cynical hostility increases whereas sense of coherence decreases the odds for current suicidal thoughts: A cross‐sectional study of the general adult population sample
Source: Health Sci Rep. 2023 Jul 28;6(8):e1464. doi: 10.1002/hsr2.1464 (PMC10382655; doi:10.1002/hsr2.1464)
Supplement: Supplementary file 1 — Supporting information. [file HSR2-6-e1464-s001.docx]

Supplementary table 1. Cynical hostility (Cook-Medley) items by suicidality (two direct questions).

|  | Suicidality | No suicidality | p |
| --- | --- | --- | --- |
| Cynical hostility, items | mean (SD) | mean (SD) |  |
| I am sure that most people do not have problems with lying for their own good. | 1.8 (0.78) | 2.52 (0.76) | 0.002 |
| Most people are good and honest mainly because they are afraid to get caught. | 2.67 (0.72) | 2.73 (0.78) | 0.623 |
| Most people are ready to use any means, also dishonest ones, in order to gain benefits. | 2.20 (0.78) | 2.82 (0.72) | 0.003 |
| I often think what could be the real reasons when others do something for my benefit. | 2.33 (0.82) | 3.28 (0.72) | <0.001 |
| Nobody cares much about what happens to somebody else. | 2.93 (0.70) | 3.48 (0.57) | 0.004 |
| It is better not to trust anyone. | 3.27 (0.88) | 3.66 (0.66) | 0.029 |
| Most people make friends because they think friends can be useful for their purposes. | 2.73 (0.70) | 3.45 (0.72) | <0.001 |
| Most people would not want to go through the trouble to help other people. | 2.53 (0.64) | 3.34 (0.72) | <0.001 |

Abbreviation: SD = standard deviation.

Supplementary table 2. Principal component analysis of cynical hostility items together with the HSCL-25 item on suicidal thoughts.*

|  | Factors | |  |
| --- | --- | --- | --- |
| Cynical hostility, items | Factor 2 | Factor 1 | Sum square |
| Most people would not want to go through the trouble to help other people. | 0.709 | 0.332 | 0.613 |
| It is better not to trust anyone. | 0.698 | 0.320 | 0.590 |
| Most people make friends because they think friends can be useful for their purposes. | 0.697 | 0.347 | 0.607 |
| Nobody cares much about what happens to somebody else. | 0.666 | 0.342 | 0.560 |
| I often think what could be the real reasons when others do something for my benefit. | 0.480 | 0.349 | 0.352 |
| Most people are ready to use any means, also dishonest ones, in order to gain benefits. | 0.356 | 0.788 | 0.748 |
| I am sure that most people do not have problems with lying for their own good. | 0.304 | 0.660 | 0.528 |
| Most people are good and honest mainly because they are afraid to get caught. | 0.314 | 0.425 | 0.279 |
| HSCL-25, item 23 (suicidal thoughts) | -0.176 | -0.094 | 0.040 |
| Sum square | 2.499 | 1.818 | 4.317 |

*Cronbach´s alpha = 0.86. Abbreviation: HSCL-25 = 25-item Hopkins Symptom Checklist.

Supplementary table 3. Principal component analysis of cynical hostility items together with one direct question on suicidality (suicidal ideation).*

|  | Factors | |  |
| --- | --- | --- | --- |
| Cynical hostility, items | Factor 2 | Factor 1 | Sum square |
| It is better not to trust anyone. | 0.647 | 0.200 | 0.459 |
| Most people would not want to go through the trouble to help other people. | 0.643 | 0.227 | 0.465 |
| I often think what could be the real reasons when others do something for my benefit. | 0.580 | 0.232 | 0.390 |
| Most people make friends because they think friends can be useful for their purposes. | 0.577 | 0.514 | 0.597 |
| Nobody cares much about what happens to somebody else. | 0.507 | 0.308 | 0.352 |
| I am sure that most people do not have problems with lying for their own good. | 0.493 | 0.380 | 0.387 |
| Most people are good and honest mainly because they are afraid to get caught. | 0.068 | 0.995 | 0.995 |
| Most people are ready to use any means, also dishonest ones, in order to gain benefits. | 0.474 | 0.476 | 0.451 |
| Suicidal ideation (one direct question) | -0.564 | 0.010 | 0.318 |
| Sum square | 2.548 | 1.865 | 4.413 |

*Cronbach´s alpha = 0.80.

Supplementary table 4. Principal component analysis of cynical hostility items together with two direct questions on suicidality.*

|  | Factors | |  |
| --- | --- | --- | --- |
| Cynical hostility, items | Factor 2 | Factor 1 | Sum square |
| It is better not to trust anyone. | 0.661 | 0.187 | 0.472 |
| Most people would not want to go through the trouble to help other people. | 0.657 | 0.215 | 0.478 |
| Most people make friends because they think friends can be useful for their purposes. | 0.586 | 0.504 | 0.597 |
| I often think what could be the real reasons when others do something for my benefit. | 0.573 | 0.223 | 0.378 |
| Nobody cares much about what happens to somebody else. | 0.512 | 0.299 | 0.351 |
| I am sure that most people do not have problems with lying for their own good. | 0.498 | 0.371 | 0.386 |
| Most people are good and honest mainly because they are afraid to get caught. | 0.471 | 0.468 | 0.441 |
| Most people are ready to use any means, also dishonest ones, in order to gain benefits. | 0.085 | 0.994 | 0.995 |
| Suicidality (two direct questions) | -0.495 | -0.014 | 0.245 |
| Sum square | 2.524 | 1.820 | 4.344 |

*Cronbach´s alpha = 0.74.
